# Subject Index Volume 14

**DOI:** 10.1111/jdi.14122

**Published:** 2023-11-30

α‐Cells

Newly discovered knowledge pertaining to glucagon and its clinical applications, Kawamori 829–837.

β‐Cell

Metabolic signatures of β‐cell destruction in type 1 diabetes, Noso 48–57.

β‐Cell imaging

Noninvasive evaluation of donor and native pancreases following simultaneous pancreas–kidney transplantation using positron emission tomography/computed tomography, Murakami 1187–1191.

β‐Cell mass

Noninvasive evaluation of donor and native pancreases following simultaneous pancreas–kidney transplantation using positron emission tomography/computed tomography, Murakami 1187–1191.

25‐OH‐vitamin D

Association of vitamin D status with all‐cause mortality and outcomes among Chinese individuals with diabetic foot ulcers, Weiwei Tang 122–131.

3 Screen ICA

Comparison of positive rates between glutamic acid decarboxylase antibodies and ElisaRSR™ 3 Screen ICA™ in recently obtained sera from patients who had been previously diagnosed with slowly progressive type 1 diabetes, Takehana 856–863.

Improving diagnostic accuracy of 3 Screen ICA ELISA kit in the identification of Japanese type 1 diabetes, Kawasaki 1081–1091.

*ABCC8*

Targeted gene panel analysis of Japanese patients with maturity‐onset diabetes of the young‐like diabetes mellitus: Roles of inactivating variants in the *ABCC8* and insulin resistance genes, Yorifuji 387–403.

Acute‐to‐chronic glycemic ratio

Association of acute‐to‐chronic glycemic ratio and outcomes in patients with COVID‐19 and undiagnosed diabetes mellitus: A retrospective nationwide cohort study, Uchihara 623–629.

Adenosine monophosphate deaminase

Adenosine monophosphate deaminase in the endoplasmic reticulum–mitochondria interface promotes mitochondrial Ca^2+^ overload in type 2 diabetes rat hearts, Osanami 560–569.

Adiponectin

Sex differences in predictive factors for onset of type 2 diabetes in Japanese individuals: A 15‐year follow‐up study, Yoshimoto 37–47.

Administrative claims data

Impact of coexisting diabetes on survival and risk of developing second primary cancer in diabetes patients receiving drug therapy: A multicenter retrospective cohort study of patients with cancer in Japan, Kuwabara 329–338.

Adverse pregnancy outcomes

Maternal uric acid levels and risk of gestational diabetes mellitus: A systematic review and dose–response meta‐analysis of cohort studies including 105,380 participants, Nikparast 973–984.

Affinity

Bivalent GAD autoantibody ELISA improves clinical utility and risk prediction for adult autoimmune diabetes, Kawasaki 570–581.

Amino acid

Characteristic changes in plasma glutamate levels and free amino acid profiles in Japanese patients with type 1 diabetes mellitus, Kawamori 111–121.

Blockade of glucagon increases muscle mass and alters fiber type composition in mice deficient in proglucagon‐derived peptides, Ueno 1045–1055.

Amino acids

Newly discovered knowledge pertaining to glucagon and its clinical applications, Kawamori 829–837.

Animal model

Contribution of animal models to diabetes research: Its history, significance, and translation to humans, Yagihashi 1015–1037.

Anti‐islet autoantibodies

Clinical signs of type 1 diabetes are associated with type 2 diabetes marker transcription factor 7‐like 2 polymorphism, Ergur 221–229.

Antiretroviral therapy

New‐onset type 1 diabetes and Graves' disease after antiretroviral therapy in a patient with human immunodeficiency virus infection, Taguchi 489–493.

Aortic endothelial dysfunction

Luseogliflozin and caloric intake restriction increase superoxide dismutase 2 expression, promote antioxidative effects, and attenuate aortic endothelial dysfunction in diet‐induced obese mice, Kawade 548–559.

Application

Validation and application of the 2019 International Working Group on the Diabetic Foot risk stratification for diabetic foot in Chinese patients, Hu 893–901.

Atherosclerosis

Sex differences in the association between visceral adipose tissue and atherosclerosis in type 2 diabetes patients with normal bodyweight: A study in a Chinese population, Xu 92–101.

Weight loss improves inflammation by T helper 17 cells in an obese patient with psoriasis at high risk for cardiovascular events, Maezawa 1,136–1,139.

Atherosclerotic cardiovascular disease

Machine learning for the prediction of atherosclerotic cardiovascular disease during 3‐year follow up in Chinese type 2 diabetes mellitus patients, Ding 1289–1302.

Autoantibodies

Comparing the clinical significance and antigen specificity of insulinoma‐associated antigen‐2 autoantibodies between radioimmunoassay and enzyme‐linked immunosorbent assay in Japanese patients with type 1 diabetes, Kawasaki 58–66.

Bivalent GAD autoantibody ELISA improves clinical utility and risk prediction for adult autoimmune diabetes, Kawasaki 570–581.

Autoantigen

Comparing the clinical significance and antigen specificity of insulinoma‐associated antigen‐2 autoantibodies between radioimmunoassay and enzyme‐linked immunosorbent assay in Japanese patients with type 1 diabetes, Kawasaki 58–66.

Autophagy

Exosomal circHIPK3 derived from umbilical cord‐derived mesenchymal stem cells enhances skin fibroblast autophagy by blocking miR‐20b5p/ULK1/Atg13 axis, Qiu 1344–1355.

Autoimmunity

Graft failure after allogeneic islet transplantation in a patient with type 1 diabetes and a high anti‐glutamic acid decarboxylase antibody titer, Kodani 725–729.

Automated insulin delivery

Diabetes technology including automated insulin delivery systems to manage hyperglycemia in a failing pancreatic graft: Case series of people with type 1 diabetes and a pancreas kidney or pancreas‐only transplant, Stathi 917–920.

Insulinoma‐associated antigen‐2

Comparing the clinical significance and antigen specificity of insulinoma‐associated antigen‐2 autoantibodies between radioimmunoassay and enzyme‐linked immunosorbent assay in Japanese patients with type 1 diabetes, Kawasaki 58–66.

Balance

Immediate effect of textured insoles on the balance in patients with diabetic neuropathy, Alaee 435–440.

Bangladesh

Baseline prevalence of hyperglycemia and its predictors among community clinic users of a selected rural area of Bangladesh: A cross‐sectional study using the WHO PEN Protocol 1, Faruque 1368–1377.

BASP1

BASP1 promotes high glucose‐induced endothelial apoptosis in diabetes via activation of EGFR signaling, Sun 535–547.

BDI‐II

Validity of the short‐form five‐item Problem Area in Diabetes questionnaire as a depression screening tool in type 2 diabetes mellitus patients, Tay 1128–1135.

Behavioral change stage

Behavioral change stage might moderate the impact of multifaceted interventions on non‐attendance from medical care among patients with type 2 diabetes: The Japan Diabetes Outcome Intervention Trial‐2 Large‐Scale Trial007 (J‐DOIT2‐LT007), Bouchi 907–916.

Beta cells

Signaling pathways that regulate adaptive β‐cell proliferation for the treatment of diabetes, Shirakawa 735–740.

Bleeding complication

Factors associated with bleeding complications in patients with coronavirus disease 2019 admitted to intensive care units: A multicenter retrospective cohort study, Imai 1312–1317.

Bone mineral density

Association between muscle mass, bone mineral density and osteoporosis in type 2 diabetes, Pan 351–358.

Bullous pemphigoid

Risk heterogeneity of bullous pemphigoid among dipeptidyl peptidase‐4 inhibitors: A population‐based cohort study using Japanese Latter‐Stage Elderly Healthcare Database, Harano 756–766.

Cardiac dysfunction

Association between cortisol and left ventricular diastolic dysfunction in patients with diabetes mellitus, Sagara 344–350.

Cardiometabolic risk factors

Cardiometabolic risk reductions in patients with type 2 diabetes mellitus newly treated with a sodium–glucose cotransporter 2inhibitor versus a dipeptidyl peptidase‐4 inhibitor: A real‐world administrative database study in Japan, Kashiwagi 404–416.

Cardiovascular autonomic neuropathy

Urinary epidermal growth factor levels correlate with cardiovascular autonomic neuropathy indices in adults with type 1 diabetes, Lin 1183–1186.

Cardiovascular disease

Real‐world risk of cardiovascular diseases in patients with type 2 diabetes associated with sodium–glucose cotransporter 2 inhibitors in comparison with metformin: A propensity score‐matched model analysis in Japan, Horii 1262–1267.

Cardiovascular diseases

Empagliflozin is associated with lower risk of cardiovascular events and all‐cause mortality in routine care in East Asia: Results from the EMPRISE study, Kim 417–428.

Associations of polyneuropathy with risk of all‐cause and cardiovascular mortality, cardiovascular disease events stratified by diabetes status, Kim 1279–1288.

Cardiovascular events

Glycated hemoglobin variability and the risk of cardiovascular events in patients with prediabetes and type 2 diabetes mellitus: A post‐hoc analysis of a prospective and multicenter study, Manosroi 1391–1400.

Cardiovascular health metrics

Association of cardiovascular health metrics with annual incidence of prediabetes or diabetes: Analysis of a nationwide real‐world database, Okada 452–462.

Carotid atherosclerosis

Carotid atherosclerosis: An independent risk factor for small fiber nerve dysfunction inpatients with type 2 diabetes mellitus, Guo 289–296.

CBT

Type 1 diabetes mellitus after cord blood transplantation from an unrelated donor with a disease‐sensitive haplotype, Matsumoto 344–347.

Claims analysis

Patient referral flow between physician and ophthalmologist visits for diabetic retinopathy screening among Japanese patients with diabetes: A retrospective cross‐sectional cohort study using the National Database, Ihana‐Sugiyama 883–892.

Claims database

Association of dipeptidyl peptidase‐4 inhibitor use and risk of pancreatic cancer in individuals with diabetes in Japan, Kubota 67–74.

Coexisting diabetes

Impact of coexisting diabetes on survival and risk of developing second primary cancer in diabetes patients receiving drug therapy: A multicenter retrospective cohort study of patients with cancer in Japan, Kuwabara 329–338.

Cognitive function

Cognitive protection of incretin‐based therapies in patients with type 2 diabetes mellitus: A systematic review and meta‐analysis based on clinical studies, Chai 864–873.

Predicting three categories of Dementia Assessment Sheet for Community‐based Integrated Care System 8‐items score‐based glycemic targets using the number of animal names recalled, Hiiragi 1121–1127.

Commentary

New mechanism of metformin action mediated by lysosomal presenilin enhancer 2, Sugawara 12–14.

Pancreatic islet transplantation in type 1diabetes: Current state and future perspectives, Kuo 183–185.

Current understanding of imeglimin action on pancreatic β‐cells: Involvement of mitochondria and endoplasmic reticulum homeostasis, Fauzi 186–188.

Glucagon‐like peptide‐1 therapy for youth with type 2 diabetes, Cho 362–363.

Promoting pancreatic β‐cell proliferation: A powerful key for realizing regenerative therapy for diabetes, Shimaya 522–524.

Neuroprotective properties of DPP‐4i: A therapeutic target for dementia prevention in elderly diabetic patients?, Umemura 525–527.

Can lipophilic pollutants in adipose tissue explain weight change‐related risk in type 2 diabetes mellitus?, Lee 528–530.

Which comes first in type 1 diabetes: Autoimmunity or dysglycemia?, Ikegami 645–647.

Commentary on glucose reduction in type 2 diabetes (GRADE), Lee 741–743.

Gut as a source of new β‐cells and Segi's cap, Yagihashi 838–840.

Is a low‐carbohydrate, high‐fat diet feasible for people with type 2 diabetes and nonalcoholic fatty liver disease?, Yen 930–932.

Is caloric restriction enough to increase longevity? Fasting and circadian alignment, Hamamoto 933–935.

Impact of the angiotensin receptor‐neprilysin inhibitor in clinical diabetes management: Potential benefits and pitfalls, Kato 1038–1040.

Serine supplementation: Is it a new option for the treatment of diabetic polyneuropathy?, Mizukami 1,157–1,159.

Delineating the transcriptional atlas for impaired insulin secretion: A window into type 2 diabetes pathophysiology, Li 1231–1233.

Epigenetic regulation of core genes linking to diabetic nephropathy progression: Lesson from Finn Diane type 1 diabetes study, Makino 1341–1343.

Confectionery intake

Sleep duration and food intake in people with type 2 diabetes mellitus and factors affecting confectionery intake, Akiyama 716–724.

Congenital generalized

A subtype of laminopathies: Generalized lipodystrophy‐associated progeroid syndrome caused by LMNA gene c.29C > T mutation, Huang 1221–1225.

Continuous glucose monitoring

Factors associated with hemoglobin glycation index in adults with type 1 diabetes mellitus: The FGM‐Japan study, Sakane 582–590.

Association between low‐carbohydrate diets and continuous glucose monitoring‐derived time in ranges, Osugi 659–668.

Corneal stromal‐epithelial nerve penetration sites

Type 2 diabetes influences intraepithelial corneal nerve parameters and corneal stromal‐epithelial nerve penetration sites, Machet 591–601.

COVID‐19

Impact of the COVID‐19 pandemic on the glycemic control, eating habits, and body compositions of people with diabetes mellitus: A retrospective longitudinal observational study, Sawada 321–328.

Association of acute‐to‐chronic glycemic ratio and outcomes in patients with COVID‐19 and undiagnosed diabetes mellitus: A retrospective nationwide cohort study, Uchihara 623–629.

Impact of the COVID‐19 pandemic on the glycemic control in people with diabetes mellitus: A retrospective cohort study, Ohkuma 985–993.

Factors associated with bleeding complications in patients with coronavirus disease 2019 admitted to intensive care units: A multicenter retrospective cohort study, Imai 1312–1317.

Daidzein

Association between equol producers and type 2 diabetes mellitus among Japanese older adults, Hamaura 707–715.

Deep vein thrombosis

A case of fulminant type 1 diabetes and protein C deficiency complicated by deep vein thrombosis, Kohata 1005–1008.

Dehydroepiandrosterone

Low serum dehydroepiandrosterone levels are associated with diabetic retinopathy in patients with type 2 diabetes mellitus, Zhang 675–685.

Dementia

Association between diabetes mellitus and post‐stroke cognitive impairment, Lee 6–11.

Dementia Assessment Sheet for Community‐based Integrated Care System8‐items

Predicting three categories of Dementia Assessment Sheet for Community‐based Integrated Care System 8‐items score‐based glycemic targets using the number of animal names recalled, Hiiragi 1121–1127.

Depression

Validity of the short‐form five‐item Problem Area in Diabetes questionnaire as a depression screening tool in type 2 diabetes mellitus patients, Tay 1128–1135.

Depressive symptoms

Validity of the short‐form five‐item Problem Area in Diabetes questionnaire as a depression screening tool in type 2 diabetes mellitus patients, Tay 1128–1135.

Diabetes

Increased oxidative stress caused by impaired mitophagy aggravated liver ischemia and reperfusion injury in diabetic mice, Zhijun 28–36.

Interaction among dietary n‐3 and n‐6 polyunsaturated fatty acid intake, fatty acid desaturase 2 genetic variants, and low‐density lipoprotein cholesterol levels in type 2 diabetes patients, Huang 297–308.

Value of machine learning algorithms for predicting diabetes risk: A subset analysis from a real‐world retrospective cohort study, Mao 309–320.

Impact of the COVID‐19 pandemic on the glycemic control, eating habits, and body compositions of people with diabetes mellitus: A retrospective longitudinal observational study, Sawada 321–328.

Influence of hepatitis B virus on the prevalence of diabetes complications in patients with type 2 diabetes, Liu 429–434.

Association of cardiovascular health metrics with annual incidence of prediabetes or diabetes: Analysis of a nationwide real‐world database, Okada 452–462.

BASP1 promotes high glucose‐induced endothelial apoptosis in diabetes via activation of EGFR signaling, Sun 535–547.

Behavioral change stage might moderate the impact of multifaceted interventions on non‐attendance from medical care among patients with type 2 diabetes: The Japan Diabetes Outcome Intervention Trial‐2 Large‐Scale Trial007 (J‐DOIT2‐LT007), Bouchi 907–916.

Role of glycosylphosphatidylinositol‐anchored high‐density lipoprotein binding protein 1 in hypertriglyceridemia and diabetes, Kurooka 1148–1156.

Factors associated with bleeding complications in patients with coronavirus disease 2019 admitted to intensive care units: A multicenter retrospective cohort study, Imai 1312–1317.

Diabetes Care

Toward better diabetes care: Exploration and implementation, Sheu 640–644.

Diabetes education hospitalization program

A 7 day inpatient diabetes education program improves quality of life and glycemic control 12 months after discharge, Kurita 811–820.

Diabetes mellitus

Association between diabetes mellitus and post‐stroke cognitive impairment, Lee 6–11.

Prevalence and predictors of clinical inertia inpatients with type 2 diabetes who were treated with a single oral antidiabetic drug, Suzuki 81–91.

Diabetes mellitus impacts on expression of DNA mismatch repair protein PMS2 and tumor microenvironment in pancreatic ductal adenocarcinoma, Pan 132–144.

Proportion of subsequent clinic visits among persons without regular clinic visits who were screened as having hyperglycemia: A retrospective cohort study, Matsumura 695–706.

Association between equol producers and type 2 diabetes mellitus among Japanese older adults, Hamaura 707–715.

Effects of switching from liraglutide to semaglutide or dulaglutide in patients with type 2 diabetes: A randomized controlled trial, Iijima 774–781.

Effect of a reused insulin needle remaining in a patient's body, Qiao 821–823.

Contribution of animal models to diabetes research: Its history, significance, and translation to humans, Yagihashi 1015–1037.

miR‐31 ameliorates type 2 diabetic vascular damage through up‐regulation of hypoxia‐inducible factor‐1α/vascular endothelial growth factor‐A, Fu 1070–1080.

Predicting three categories of Dementia Assessment Sheet for Community‐based Integrated Care System 8‐items score‐based glycemic targets using the number of animal names recalled, Hiiragi 1121–1127.

Effectiveness of countermeasure for polypharmacy by multidisciplinary team review in patients with diabetes mellitus, Nishida 1202–1208.

A subtype of laminopathies: Generalized lipodystrophy‐associated progeroid syndrome caused by LMNA gene c.29C > T mutation, Huang 1221–1225.

Associations of polyneuropathy with risk of all‐cause and cardiovascular mortality, cardiovascular disease events stratified by diabetes status, Kim 1279–1288.

Diabetes mellitus, type 2

Changes in prescription patterns and doses of oral antidiabetic drugs in Japanese patients with type 2 diabetes (JDDM70), Shirabe 75–80.

Comparative efficacy of different eating patterns in the management of type 2 diabetes and prediabetes: An arm‐based Bayesian network meta‐analysis, Zeng 263–288.

Cardiometabolic risk reductions in patients with type 2 diabetes mellitus newly treated with a sodium–glucose cotransporter 2inhibitor versus a dipeptidyl peptidase‐4 inhibitor: A real‐world administrative database study in Japan, Kashiwagi 404–416.

Low serum dehydroepiandrosterone levels are associated with diabetic retinopathy in patients with type 2 diabetes mellitus, Zhang 675–685.

Diabetes technology

Diabetes technology including automated insulin delivery systems to manage hyperglycemia in a failing pancreatic graft: Case series of people with type 1 diabetes and a pancreas kidney or pancreas‐only transplant, Stathi 917–920.

Diabetes treatment

Change in fatty acid composition of plasma triglyceride caused by a 2 week comprehensive risk management for diabetes: A prospective observational study of type 2 diabetes patients with supercritical fluid chromatography/mass spectrometry‐based semi‐target lipidomic analysis, Taya 102–110.

Diabetes treatment‐related quality of life

A 7 day inpatient diabetes education program improves quality of life and glycemic control 12 months after discharge, Kurita 811–820.

Diabetes type 2

Safety and effectiveness of dulaglutide 0.75 mg in Japanese patients with type 2 diabetes in real‐world clinical practice: 36 month post‐marketing observational study, Chin 247–258.

Diabetic cardiomyopathy

Adenosine monophosphate deaminase in the endoplasmic reticulum–mitochondria interface promotes mitochondrial Ca^2+^ overload in type 2 diabetes rat hearts, Osanami 560–569.

Diabetic complications

Progress in genetics of type 2 diabetes and diabetic complications, Shojima 503–515.

Diabetic distal symmetric polyneuropathy

Ultra‐high performance liquid chromatography coupled to tandem mass spectrometry‐based metabolomics study of diabetic distal symmetric polyneuropathy, Xu 1110–1120.

Diabetic ketoacidosis

A case of fulminant type 1 diabetes and protein C deficiency complicated by deep vein thrombosis, Kohata 1,005–1,008.

Diabetic ketoacidosis due to a sensor defect of FreeStyleLibre: A case report, Kogai 1321–1324.

Diabetic foot

Validation and application of the 2019 International Working Group on the Diabetic Foot risk stratification for diabetic foot in Chinese patients, Hu 893–901.

Diabetic foot ulcer

Non‐classical monocytes frequency and serum vitamin D_3_ levels are linked to diabetic foot ulcer associated with peripheral artery disease, Hammad 1192–1201.

Diabetic foot ulcers

Association of vitamin D status with all‐cause mortality and outcomes among Chinese individuals with diabetic foot ulcers, Weiwei Tang 122–131.

Diabetic kidney disease

Suramin prevents the development of diabetic kidney disease by inhibiting NLRP3inflammasome activation in KK‐Ay mice, Oda 205–220.

Polar vasculosis is associated with better kidney outcome in type 2 diabetes with biopsy‐proven diabetic kidney disease: A multicenter cohort study, Shimizu 1268–1278.

Diabetic nephropathy.

Progesterone levels associated with proteinuria in male diabetes mellitus patients: A cross‐sectional retrospective study, Liu 669–674.

LncRNA SNHG1 knockdown inhibits hyperglycemia induced ferroptosis via miR‐16‐5p/ACSL4 axis to alleviate diabetic nephropathy, Fang 1056–1069.

Diabetic peripheral neuropathic pain

Sinomenine alleviates diabetic peripheral neuropathic pain through inhibition of the inositol‐requiring enzyme 1 alpha–X‐box binding protein 1 pathway by downregulating prostaglandin‐endoperoxide synthase 2, Chen 364–375.

Diabetic peripheral neuropathy

Type 2 diabetes influences intraepithelial corneal nerve parameters and corneal stromal‐epithelial nerve penetration sites, Machet 591–601.

Diabetic retinopathy

Low serum dehydroepiandrosterone levels are associated with diabetic retinopathy in patients with type 2 diabetes mellitus, Zhang 675–685.

Patient referral flow between physician and ophthalmologist visits for diabetic retinopathy screening among Japanese patients with diabetes: A retrospective cross‐sectional cohort study using the National Database, Ihana‐Sugiyama 883–892.

Diagnostic marker

Diagnostic ability using fatty liver and metabolic markers for metabolic‐associated fatty liver disease stratified by metabolic/glycemic abnormalities, Okada 463–478.

Diagnostic accuracy

Improving diagnostic accuracy of 3 Screen ICA ELISA kit in the identification of Japanese type 1 diabetes, Kawasaki 1081–1091.

Diagnostic screening programs

Proportion of subsequent clinic visits among persons without regular clinic visits who were screened as having hyperglycemia: A retrospective cohort study, Matsumura 695–706.

Diet

Fermented soybean foods and diabetes, Hashimoto 1329–1340.

Dipeptidyl peptidase‐4 inhibitor

Association of dipeptidyl peptidase‐4 inhibitor use and risk of pancreatic cancer in individuals with diabetes in Japan, Kubota 67–74.

Association of dipeptidyl peptidase‐4 inhibitor use and risk of pancreatic cancer in individuals with diabetes in Japan, Kubota 67–74.

Disease‐sensitive haplotype

Type 1 diabetes mellitus after cord blood transplantation from an unrelated donor with a disease‐sensitive haplotype, Matsumoto 344–347.

Distress

Validity of the short‐form five‐item Problem Area in Diabetes questionnaire as a depression screening tool in type 2 diabetes mellitus patients, Tay 1128–1135.

DKA

*MNX1* mutations causing neonatal diabetes: Review of the literature and report of a case with extra‐pancreatic congenital defects presenting in severe diabetic ketoacidosis, Aly 516–521.

DM

Exosomal circHIPK3 derived from umbilical cord‐derived mesenchymal stem cells enhances skin fibroblast autophagy by blocking miR‐20b5p/ULK1/Atg13 axis, Qiu 1344–1355.

DPP‐4 inhibitor

Risk heterogeneity of bullous pemphigoid among dipeptidyl peptidase‐4 inhibitors: A population‐based cohort study using Japanese Latter‐Stage Elderly Healthcare Database, Harano 756–766.

Drug adverse reaction

Risk heterogeneity of bullous pemphigoid among dipeptidyl peptidase‐4 inhibitors: A population‐based cohort study using Japanese Latter‐Stage Elderly Healthcare Database, Harano 756–766.

Dulaglutide

Safety and effectiveness of dulaglutide 0.75 mg in Japanese patients with type 2 diabetes in real‐world clinical practice: 36 month post‐marketing observational study, Chin 247–258.

Effects of switching from liraglutide to semaglutide or dulaglutide in patients with type 2 diabetes: A randomized controlled trial, Iijima 774–781.

Duration of diabetes

A 7 day inpatient diabetes education program improves quality of life and glycemic control 12 months after discharge, Kurita 811–820.

Editorial

Prospects of stem cell therapy for diabetic microvascular complications, Naruse 3–5.

Mechanisms and the strategy for remission of type 2 diabetes mellitus, Chang 351–353.

A novel view of the insulin signaling pathway based on prediction of protein structure by the AI platform AlphaFold, Sato 635–639.

Sarcopenia: Loss of mighty armor against frailty and aging, Sasako 1145–1147.

EGFR

BASP1 promotes high glucose‐induced endothelial apoptosis in diabetes via activation of EGFR signaling, Sun 535–547.

Electrocardiogram abnormalities

Electrocardiogram abnormalities and renal impairment in patients with type 2 diabetes mellitus: A healthcare facilities‐based cross‐sectional study in Dang district of Nepal, Khanal 602–613.

Endothelial growth factor

Urinary epidermal growth factor levels correlate with cardiovascular autonomic neuropathy indices in adults with type 1 diabetes, Lin 1183–1186.

Equol

Association between equol producers and type 2 diabetes mellitus among Japanese older adults, Hamaura 707–715.

Esophagogastroduodenoscopy

Association of glucagon‐like peptide‐1 receptor agonist treatment with gastric residue in an esophagogastroduodenoscopy, Kobori 767–773.

Estimated glomerular filtration rate

Independent risk factors of rapid glomerular filtration rate decline in patients with type 2 diabetes with preserved kidney function and normoalbuminuria: A multicenter cohort study, Hirano 874–882.

Exo‐circHIPK3

Exosomal circHIPK3 derived from umbilical cord‐derived mesenchymal stem cells enhances skin fibroblast autophagy by blocking miR‐20b5p/ULK1/Atg13 axis, Qiu 1344–1355.

Exosome

LINC01705 derived from adipocyte exosomes regulates hepatocyte lipid accumulation via an miR‐552‐3p/LXR axis, Qiu 1160–1171.

Exploration

Toward better diabetes care: Exploration and implementation, Sheu 640–644.

*FADS1*

Interaction among dietary n‐3 and n‐6 polyunsaturated fatty acid intake, fatty acid desaturase 2 genetic variants, and low‐density lipoprotein cholesterol levels in type 2 diabetes patients, Huang 297–308.

*FADS2*

Interaction among dietary n‐3 and n‐6 polyunsaturated fatty acid intake, fatty acid desaturase 2 genetic variants, and low‐density lipoprotein cholesterol levels in type 2 diabetes patients, Huang 297–308.

Fasting blood glucose

Association between serum gamma glutamyl transferase and fasting blood glucose in Chinese people: A 6‐year follow‐up study, Teng 339–343.

Fermented food

Fermented soybean foods and diabetes, Hashimoto 1,329–1,340.

Fibrosis

Linagliptin ameliorated cardiac fibrosis and restored cardiomyocyte structure in diabetic mice associated with the suppression of necroptosis, Adhikari 844–855.

Flash glucose monitoring

Frequent scanning using flash glucose monitoring contributes to better glycemic control in children and adolescents with type 1 diabetes, Urakami 185–190.

Impact of flash glucose monitoring on glycemic control varies with the age and residual β‐cell function of patients with type 1 diabetes mellitus, Zhang 552–559.

Follow‐up study

Association between serum gamma glutamyl transferase and fasting blood glucose in Chinese people: A 6‐year follow‐up study, Teng 339–343.

Foot orthosis

Immediate effect of textured insoles on the balance in patients with diabetic neuropathy, Alaee 435–440.

GAD

Bivalent GAD autoantibody ELISA improves clinical utility and risk prediction for adult autoimmune diabetes, Kawasaki 570–581.

Gamma‐glutamyl transferase

Association between serum gamma glutamyl transferase and fasting blood glucose in Chinese people: A 6‐year follow‐up study, Teng 339–343.

Gastric residue

Association of glucagon‐like peptide‐1 receptor agonist treatment with gastric residue in an esophagogastroduodenoscopy, Kobori 767–773.

Gender

Progesterone levels associated with proteinuria in male diabetes mellitus patients: A cross‐sectional retrospective study, Liu 669–674.

Gender difference

Sex differences in predictive factors for onset of type 2 diabetes in Japanese individuals: A 15‐year follow‐up study, Yoshimoto 37–47.

Gestational diabetes

Risk of fetal undergrowth in the management of gestational diabetes mellitus in Japan, Kawasaki, 614–622.

Gestational diabetes mellitus

Maternal uric acid levels and risk of gestational diabetes mellitus: A systematic review and dose–response meta‐analysis of cohort studies including 105,380 participants, Nikparast 973–984.

GIP

Incretin hormone responses to carbohydrate and protein/fat are preserved in adults with sulfonylurea‐treated KCNJ11 neonatal diabetes, Bowman 1378–1382.

Glicentin

A newly developed glucagon sandwich ELISA is useful for more accurate glucagon evaluation than the currently used sandwich ELISA in subjects with elevated plasma proglucagon‐derived peptide levels, Kobayashi 648–658.

Global longitudinal strain

Association of the triglyceride‐glucose index with subclinical left ventricular dysfunction in type 2 diabetes mellitus patients: A retrospective cross‐sectional study, Sun 953–960.

GLP‐1

Incretin hormone responses to carbohydrate and protein/fat are preserved in adults with sulfonylurea‐treated KCNJ11 neonatal diabetes, Bowman 1378–1382.

Glucagon

Characteristic changes in plasma glutamate levels and free amino acid profiles in Japanese patients with type 1 diabetes mellitus, Kawamori 111–121.

A newly developed glucagon sandwich ELISA is useful for more accurate glucagon evaluation than the currently used sandwich ELISA in subjects with elevated plasma proglucagon‐derived peptide levels, Kobayashi 648–658.

Newly discovered knowledge pertaining to glucagon and its clinical applications, Kawamori 829–837.

Blockade of glucagon increases muscle mass and alters fiber type composition in mice deficient in proglucagon‐derived peptides, Ueno 1045–1055.

Robust increase in glucagon secretion after oral protein intake, but not after glucose or lipid intake in Japanese people without diabetes, Ichikawa 1172–1174.

Glucagon‐like peptide‐1

Glucose‐dependent insulinotropic polypeptide and glucagon‐like peptide‐1 secretion in humans: Characteristics and regulation, Alsalim 354–361.

Stimulatory effect of imeglimin on incretin secretion, Yingyue 746–755.

Glucagon‐like peptide‐1 receptor.

Safety and effectiveness of dulaglutide 0.75 mg in Japanese patients with type 2 diabetes in real‐world clinical practice: 36 month post‐marketing observational study, Chin 247–258.

Glucagon‐like peptide‐1 receptor agonist treatment

Association of glucagon‐like peptide‐1 receptor agonist treatment with gastric residue in an esophagogastroduodenoscopy, Kobori 767–773.

Glucose‐dependent insulinotropic polypeptide

Glucose‐dependent insulinotropic polypeptide and glucagon‐like peptide‐1 secretion in humans: Characteristics and regulation, Alsalim 354–361.

Glucose‐lowering drugs

Characteristics of subjects with type 2 diabetes enrolled in randomized controlled trials and non‐randomized controlled trials in Japan: A systematic review, Kadowaki 236–246.

Glucose‐lowering effect

Circulating selenoprotein P levels predict glucose‐lowering and insulinotropic effects of metformin, but not alogliptin: A post‐hoc analysis, Takeshita 230–235.

Glucose‐stimulated insulin secretion

Stimulatory effect of imeglimin on incretin secretion, Yingyue 746–755.

Glucose transporter type 4 (GLUT4)

SNARE‐binding protein synaptosomal‐associated protein of 29 kDa (SNAP29) regulates the intracellular sequestration of glucose transporter 4 (GLUT4) vesicles in adipocytes, Matsui 19–27.

Glutamate

Characteristic changes in plasma glutamate levels and free amino acid profiles in Japanese patients with type 1 diabetes mellitus, Kawamori 111–121.

Glycated hemoglobin

Association of self‐stigma with glycated hemoglobin: A single‐center, cross‐sectional study of adults with type 1 diabetes in Japan, Hamano 479–485.

Glycated hemoglobin A1c

Factors associated with hemoglobin glycation index in adults with type 1 diabetes mellitus: The FGM‐Japan study, Sakane 582–590.

Glycated hemoglobin variability

Glycated hemoglobin variability and the risk of cardiovascular events in patients with prediabetes and type 2 diabetes mellitus: A post‐hoc analysis of a prospective and multicenter study, Manosroi 1391–1400.

Glycemic control

Prevalence and predictors of clinical inertia inpatients with type 2 diabetes who were treated with a single oral antidiabetic drug, Suzuki 81–91.

Impact of the COVID‐19 pandemic on the glycemic control, eating habits, and body compositions of people with diabetes mellitus: A retrospective longitudinal observational study, Sawada 321–328.

Impact of the COVID‐19 pandemic on the glycemic control in people with diabetes mellitus: A retrospective cohort study, Ohkuma 985–993.

Glycemic variability

Factors associated with hemoglobin glycation index in adults with type 1 diabetes mellitus: The FGM‐Japan study, Sakane 582–590.

Glycated hemoglobin variability and the risk of cardiovascular events in patients with prediabetes and type 2 diabetes mellitus: A post‐hoc analysis of a prospective and multicenter study, Manosroi 1391–1400.

GPIHBP1

Role of glycosylphosphatidylinositol‐anchored high‐density lipoprotein binding protein 1 in hypertriglyceridemia and diabetes, Kurooka 1148–1156.

Grip strength

Resistin G–A haplotype at SNP‐420/−358 is associated with the latent sarcopenic obesity index in the toon genome study, Ikeda 686–694.

Gut microbiota

Fermented soybean foods and diabetes, Hashimoto 1329–1340.

HbA1c

False hemoglobin A1c value as a result of compound heterozygotes of hemoglobin E and hemoglobin New York, Li 494–497.

HbA1c variability

Changes of HbA1c variability after the switch to a longer‐acting insulin analog in people with type 1 diabetes, Watanabe 259–262.

HbE

False hemoglobin A1c value as a result of compound heterozygotes of hemoglobin E and hemoglobin New York, Li 494–497.

Hb New York

False hemoglobin A1c value as a result of compound heterozygotes of hemoglobin E and hemoglobin New York, Li 494–497.

Healthcare quality assessment

Patient referral flow between physician and ophthalmologist visits for diabetic retinopathy screening among Japanese patients with diabetes: A retrospective cross‐sectional cohort study using the National Database, Ihana‐Sugiyama 883–892.

Health insurance claims

Effect of the Diabetic Nephropathy Aggravation Prevention Program on medical visit behavior in individuals under the municipal national health insurance, Ikeda 782–791.

Hemoglobin glycation index

Association between hemoglobin glycation index and non‐alcoholic fatty liver disease in the patients with type 2 diabetes mellitus, Wang 1303–1311.

Hemoglobin level

Independent risk factors of rapid glomerular filtration rate decline in patients with type 2 diabetes with preserved kidney function and normoalbuminuria: A multicenter cohort study, Hirano 874–882.

Hepatitis B virus

Influence of hepatitis B virus on the prevalence of diabetes complications in patients with type 2 diabetes, Liu 429–434.

HLA

Type 1 diabetes mellitus after cord blood transplantation from an unrelated donor with a disease‐sensitive haplotype, Matsumoto 344–347.

Hypoglycemia

Methadone‐induced hypoglycemia: A case report, Malboosbaf 145–146.

Local low‐frequency vibration accelerates healing of full‐thickness wounds in a hyperglycemic rat model, Haba 1356–1367.

Baseline prevalence of hyperglycemia and its predictors among community clinic users of a selected rural area of Bangladesh: A cross‐sectional study using the WHO PEN Protocol 1, Faruque 1368–1377.

Hyperglycemic crises

Pontine lesion in hyperglycemic crises: Relevance to osmotic demyelination syndrome and posterior reversible encephalopathy syndrome, Murao 486–488.

Hypoglycemic agents

Changes in prescription patterns and doses of oral antidiabetic drugs in Japanese patients with type 2 diabetes (JDDM70), Shirabe 75–80.

Prevalence and predictors of clinical inertia inpatients with type 2 diabetes who were treated with a single oral antidiabetic drug, Suzuki 81–91.

Hypoxia‐inducible factor

miR‐31 ameliorates type 2 diabetic vascular damage through up‐regulation of hypoxia‐inducible factor‐1α/vascular endothelial growth factor‐A, Fu 1070–1080.

Imeglimin

Stimulatory effect of imeglimin on incretin secretion, Yingyue 746–755.

Effect of patient characteristics on the efficacy and safety of imeglimin monotherapy in Japanese patients with type 2 diabetes mellitus: A post‐hoc analysis of two randomized, placebo‐controlled trials, Hagi 1101–1109.

Efficacy, safety and tolerability of imeglimin in patients with type 2 diabetes mellitus: A meta‐analysis of randomized controlled trials, Hagi 1246–1261.

Imeglimin‐mediated glycemic control in maternally inherited deafness and diabetes, Ishibashi 1419–1422.

Immune checkpoint inhibitor‐associated diabetes mellitus

Recovery from insulin dependence in immune checkpoint inhibitor‐associated diabetes mellitus: A case report, Okubo 147–150.

Immune‐inflammatory reconstitution syndrome

New‐onset type 1 diabetes and Graves' disease after antiretroviral therapy in a patient with human immunodeficiency virus infection, Taguchi 489–493.

Immune‐related adverse event

Recovery from insulin dependence in immune checkpoint inhibitor‐associated diabetes mellitus: A case report, Okubo 147–150.

Implementation

Toward better diabetes care: Exploration and implementation, Sheu 640–644.

Incidence

Trends in incidence rates of childhood type 1diabetes mellitus: A retrospective study in Isfahan province, Iran, Hashemipour 376–386.

Prediabetes and the risk of lung cancer incidence and mortality: A meta‐analysis, Shen 1209–1220.

Incretin‐based therapy

Cognitive protection of incretin‐based therapies in patients with type 2 diabetes mellitus: A systematic review and meta‐analysis based on clinical studies, Chai 864–873.

Incretin hormones

Incretin hormone responses to carbohydrate and protein/fat are preserved in adults with sulfonylurea‐treated KCNJ11 neonatal diabetes, Bowman 1378–1382.

Inflammasomes

Suramin prevents the development of diabetic kidney disease by inhibiting NLRP3inflammasome activation in KK‐Ay mice, Oda 205–220.

Injections subcutaneous

Effect of a reused insulin needle remaining in a patient's body, Qiao 821–823.

*INSR*

Targeted gene panel analysis of Japanese patients with maturity‐onset diabetes of the young‐like diabetes mellitus: Roles of inactivating variants in the *ABCC8* and insulin resistance genes, Yorifuji 387–403.

Insulin

SNARE‐binding protein synaptosomal‐associated protein of 29 kDa (SNAP29) regulates the intracellular sequestration of glucose transporter 4 (GLUT4) vesicles in adipocytes, Matsui 19–27.

Changes of HbA1c variability after the switch to a longer‐acting insulin analog in people with type 1 diabetes, Watanabe 259–262.

Insulin pump

Successful introduction of sensor‐augmented pump therapy in a patient with diabetes and needle phobia: A case report, Sugai 1318–1320.

Insulin resistance

Signaling pathways that regulate adaptive β‐cell proliferation for the treatment of diabetes, Shirakawa 735–740.

Insulinotropic effects

Circulating selenoprotein P levels predict glucose‐lowering and insulinotropic effects of metformin, but not alogliptin: A post‐hoc analysis, Takeshita 230–235.

Intensive lifestyle intervention

Effect of intensive lifestyle intervention on the association between weight variability and major adverse cardiovascular events in overweight or obese adults with type 2 diabetes mellitus, Zhong 441–451.

Intermittently scanned continuous glucose monitoring

Diabetic ketoacidosis due to a sensor defect of FreeStyleLibre: A case report, Kogai 1321–1324.

Intraepithelial corneal basal nerves

Type 2 diabetes influences intraepithelial corneal nerve parameters and corneal stromal‐epithelial nerve penetration sites, Machet 591–601.

Islet autoantibodies

Comparison of positive rates between glutamic acid decarboxylase antibodies and ElisaRSR™ 3 Screen ICA™ in recently obtained sera from patients who had been previously diagnosed with slowly progressive type 1 diabetes, Takehana 856–863.

Islet autoantibody

A surge in serum mucosal cytokines associated with seroconversion in children at risk for type 1 diabetes, Harrison 1092–1100.

Islets

Signaling pathways that regulate adaptive β‐cell proliferation for the treatment of diabetes, Shirakawa 735–740.

Islet transplantation

Graft failure after allogeneic islet transplantation in a patient with type 1 diabetes and a high anti‐glutamic acid decarboxylase antibody titer, Kodani 725–729.

Japan

Changes in prescription patterns and doses of oral antidiabetic drugs in Japanese patients with type 2 diabetes (JDDM70), Shirabe 75–80.

JDI Updates

Advances in insulin therapy from discovery to β‐cell replacement, Sakurai 15–18.

Updates of incretin‐related drugs for the treatment of type 2 diabetes, Araki 167–347.

Update information on type 1 diabetes in children/adolescents and adults, Miura 531–534.

Recent updates in factors associated with incidence and screening of diabetic eye disease, Sone 744–745.

The era of continuous glucose monitoring and its expanded role in type 2 diabetes, Yu 841–843.

Diet and exercise are a fundamental part of comprehensive care for type 2 diabetes, Yeh 936–939.

Updates on dyslipidemia in patients with diabetes, Ide 1041–1044.

Effects of nutrient metabolism on pancreatic β‐cell mass and function: Recent findings, Yasuda 1234–1236.

Kidney function decline

Independent risk factors of rapid glomerular filtration rate decline in patients with type 2 diabetes with preserved kidney function and normoalbuminuria: A multicenter cohort study, Hirano 874–882.

Kidney outcome

Polar vasculosis is associated with better kidney outcome in type 2 diabetes with biopsy‐proven diabetic kidney disease: A multicenter cohort study, Shimizu 1268–1278.

Laminopathies

A subtype of laminopathies: Generalized lipodystrophy‐associated progeroid syndrome caused by LMNA gene c.29C > T mutation, Huang 1221–1225.

Lipoatrophic

A subtype of laminopathies: Generalized lipodystrophy‐associated progeroid syndrome caused by LMNA gene c.29C > T mutation, Huang 1221–1225.

Large‐for‐gestational age.

Risk of fetal undergrowth in the management of gestational diabetes mellitus in Japan, Kawasaki, 614–622.

Letter to the Editor

Insulin allergy manifesting soon after COVID‐19 vaccination (BNT162b2), Komamura 498–499.

No change in small low‐density lipoprotein cholesterol levels with pemafibrate might explain the negative results of the PROMINENT trial, Hirano 630–631.

Fibroblast growth factor 21 as a candidate of a novel serum biomarker for mitochondrial diabetes, Komorita 1,009–1,010.

Linagliptin

Linagliptin ameliorated cardiac fibrosis and restored cardiomyocyte structure in diabetic mice associated with the suppression of necroptosis, Adhikari 844–855.

LINC01705

LINC01705 derived from adipocyte exosomes regulates hepatocyte lipid accumulation via an miR‐552‐3p/LXR axis, Qiu 1160–1171.

Lipid control

Impact of the COVID‐19 pandemic on the glycemic control in people with diabetes mellitus: A retrospective cohort study, Ohkuma 985–993.

Lipid load

Robust increase in glucagon secretion after oral protein intake, but not after glucose or lipid intake in Japanese people without diabetes, Ichikawa 1172–1174.

Lipidomics

Change in fatty acid composition of plasma triglyceride caused by a 2 week comprehensive risk management for diabetes: A prospective observational study of type 2 diabetes patients with supercritical fluid chromatography/mass spectrometry‐based semi‐target lipidomic analysis, Taya 102–110.

Lipodystrophy

A subtype of laminopathies: Generalized lipodystrophy‐associated progeroid syndrome caused by LMNA gene c.29C > T mutation, Huang 1221–1225.

Lipoprotein lipase

Role of glycosylphosphatidylinositol‐anchored high‐density lipoprotein binding protein 1 in hypertriglyceridemia and diabetes, Kurooka 1148–1156.

Liver ischemia and reperfusion injury

Increased oxidative stress caused by impaired mitophagy aggravated liver ischemia and reperfusion injury in diabetic mice, Zhijun 28–36.

LncRNA SNHG1

LncRNA SNHG1 knockdown inhibits hyperglycemia induced ferroptosis via miR‐16‐5p/ACSL4 axis to alleviate diabetic nephropathy, Fang 1056–1069.

Low birth weight

Risk of fetal undergrowth in the management of gestational diabetes mellitus in Japan, Kawasaki, 614–622.

Low‐carbohydrate diet

Association between low‐carbohydrate diets and continuous glucose monitoring‐derived time in ranges, Osugi 659–668.

Low‐frequency vibration

Local low‐frequency vibration accelerates healing of full‐thickness wounds in a hyperglycemic rat model, Haba 1356–1367.

Lung cancer

Prediabetes and the risk of lung cancer incidence and mortality: A meta‐analysis, Shen 1209–1220.

Machine learning

Machine learning for the prediction of atherosclerotic cardiovascular disease during 3‐year follow up in Chinese type 2 diabetes mellitus patients, Ding 1289–1,302.

Machine learning algorithms

Value of machine learning algorithms for predicting diabetes risk: A subset analysis from a real‐world retrospective cohort study, Mao 309–320.

Macrovascular complications

Influence of hepatitis B virus on the prevalence of diabetes complications in patients with type 2 diabetes, Liu 429–434.

Major adverse cardiovascular events

Effect of intensive lifestyle intervention on the association between weight variability and major adverse cardiovascular events in overweight or obese adults with type 2 diabetes mellitus, Zhong 441–451.

Predictive values of metabolic score for insulin resistance on risk of major adverse cardiovascular events and comparison with other insulin resistance indices among Chinese with and without diabetes mellitus: Results from the 4C cohort study, Pan 961–972.

Male infant

*MNX1* mutations causing neonatal diabetes: Review of the literature and report of a case with extra‐pancreatic congenital defects presenting in severe diabetic ketoacidosis, Aly 516–521.

Maternally inherited diabetes and deafness

Imeglimin‐mediated glycemic control in maternally inherited deafness and diabetes, Ishibashi 1419–1422.

Maturity‐onset diabetes of the young

Targeted gene panel analysis of Japanese patients with maturity‐onset diabetes of the young‐like diabetes mellitus: Roles of inactivating variants in the *ABCC8* and insulin resistance genes, Yorifuji 387–403.

Meal

Glucose‐dependent insulinotropic polypeptide and glucagon‐like peptide‐1 secretion in humans: Characteristics and regulation, Alsalim 354–361.

Medical nutrition therapy

Comparative efficacy of different eating patterns in the management of type 2 diabetes and prediabetes: An arm‐based Bayesian network meta‐analysis, Zeng 263–288.

Medical visit behavior

Effect of the Diabetic Nephropathy Aggravation Prevention Program on medical visit behavior in individuals under the municipal national health insurance, Ikeda 782–791.

Metabolic‐associated fatty liver disease

Diagnostic ability using fatty liver and metabolic markers for metabolic‐associated fatty liver disease stratified by metabolic/glycemic abnormalities, Okada 463–478.

Metabolic score for insulin resistance

Predictive values of metabolic score for insulin resistance on risk of major adverse cardiovascular events and comparison with other insulin resistance indices among Chinese with and without diabetes mellitus: Results from the 4C cohort study, Pan 961–972.

Efficacy, safety and tolerability of imeglimin in patients with type 2 diabetes mellitus: A meta‐analysis of randomized controlled trials, Hagi 1246–1261.

Metabolome

Metabolic signatures of β‐cell destruction in type 1 diabetes, Noso 48–57.

Imeglimin‐mediated glycemic control in maternally inherited deafness and diabetes, Ishibashi 1419–1422.

Metabolomics

Ultra‐high performance liquid chromatography coupled to tandem mass spectrometry‐based metabolomics study of diabetic distal symmetric polyneuropathy, Xu 1110–1120.

Metformin

Real‐world risk of cardiovascular diseases in patients with type 2 diabetes associated with sodium–glucose cotransporter 2 inhibitors in comparison with metformin: A propensity score‐matched model analysis in Japan, Horii 1262–1267.

Methadone

Methadone‐induced hypoglycemia: A case report, Malboosbaf 145–146.

Mild cognitive impairment

Association of stressful life events with cognitive impairment in patients with type 2 diabetes mellitus, Wang 792–800.

Mini Review

Delivering value‐based healthcare for people with diabetes in a national publicly funded health service: Lessons from Ireland and Wales, O'Donnell 925–929.

miR‐16‐5p.

LncRNA SNHG1 knockdown inhibits hyperglycemia induced ferroptosis via miR‐16‐5p/ACSL4 axis to alleviate diabetic nephropathy, Fang 1056–1069.

miR‐552‐3p

LINC01705 derived from adipocyte exosomes regulates hepatocyte lipid accumulation via an miR‐552‐3p/LXR axis, Qiu 1160–1171.

Mitochondria

Linagliptin ameliorated cardiac fibrosis and restored cardiomyocyte structure in diabetic mice associated with the suppression of necroptosis, Adhikari 844–855.

Mitochondria‐associated endoplasmic reticulum membrane

Adenosine monophosphate deaminase in the endoplasmic reticulum–mitochondria interface promotes mitochondrial Ca^2+^ overload in type 2 diabetes rat hearts, Osanami 560–569.

Mitophagy

Increased oxidative stress caused by impaired mitophagy aggravated liver ischemia and reperfusion injury in diabetic mice, Zhijun 28–36.

Monocyte subsets

Non‐classical monocytes frequency and serum vitamin D_3_ levels are linked to diabetic foot ulcer associated with peripheral artery disease, Hammad 1192–1201.

*MNX1* mutation

*MNX1* mutations causing neonatal diabetes: Review of the literature and report of a case with extra‐pancreatic congenital defects presenting in severe diabetic ketoacidosis, Aly 516–521.

Multidisciplinary team

Effectiveness of countermeasure for polypharmacy by multidisciplinary team review in patients with diabetes mellitus, Nishida 1202–1208.

Muscle strength

Associations of sleep quality with the skeletal muscle strength in patients with type 2 diabetes with poor glycemic control, Hayashi 801–810.

Musclin

Decreased serum musclin concentration is independently associated with the high prevalence of sarcopenia in Chinese middle‐elderly patients with type 2 diabetes mellitus, Fu 1412–1418.

Myocardial fibrosis

Long non‐coding ribonucleic acid zinc finger E‐box binding homeobox 1 antisense RNA 1 regulates myocardial fibrosis in diabetes through the Hippo–Yes‐associated protein signaling pathway, Wu 940–952.

Myristic acids

Change in fatty acid composition of plasma triglyceride caused by a 2 week comprehensive risk management for diabetes: A prospective observational study of type 2 diabetes patients with supercritical fluid chromatography/mass spectrometry‐based semi‐target lipidomic analysis, Taya 102–110.

National database

Effect of the Diabetic Nephropathy Aggravation Prevention Program on medical visit behavior in individuals under the municipal national health insurance, Ikeda 782–791.

Necroptosis

Linagliptin ameliorated cardiac fibrosis and restored cardiomyocyte structure in diabetic mice associated with the suppression of necroptosis, Adhikari 844–855.

Needle

Effect of a reused insulin needle remaining in a patient's body, Qiao 821–823.

Needle phobia

Successful introduction of sensor‐augmented pump therapy in a patient with diabetes and needle phobia: A case report, Sugai 1318–1320.

Neonatal diabetes

Incretin hormone responses to carbohydrate and protein/fat are preserved in adults with sulfonylurea‐treated KCNJ11 neonatal diabetes, Bowman 1378–1382.

Nonalcoholic fatty liver disease

Diagnostic ability using fatty liver and metabolic markers for metabolic‐associated fatty liver disease stratified by metabolic/glycemic abnormalities, Okada 463–478.

Association between hemoglobin glycation index and non‐alcoholic fatty liver disease in the patients with type 2 diabetes mellitus, Wang 1303–1311.

Non‐attendance

Behavioral change stage might moderate the impact of multifaceted interventions on non‐attendance from medical care among patients with type 2 diabetes: The Japan Diabetes Outcome Intervention Trial‐2 Large‐Scale Trial007 (J‐DOIT2‐LT007), Bouchi 907–916.

Observational study

Empagliflozin is associated with lower risk of cardiovascular events and all‐cause mortality in routine care in East Asia: Results from the EMPRISE study, Kim 417–428.

Office visits

Proportion of subsequent clinic visits among persons without regular clinic visits who were screened as having hyperglycemia: A retrospective cohort study, Matsumura 695–706.

Onset of type 2 diabetes

Sex differences in predictive factors for onset of type 2 diabetes in Japanese individuals: A 15‐year follow‐up study, Yoshimoto 37–47.

Opioids

Methadone‐induced hypoglycemia: A case report, Malboosbaf 145–146.

Osmotic demyelination syndrome

Pontine lesion in hyperglycemic crises: Relevance to osmotic demyelination syndrome and posterior reversible encephalopathy syndrome, Murao 486–488.

Oxidative stress

Luseogliflozin and caloric intake restriction increase superoxide dismutase 2 expression, promote antioxidative effects, and attenuate aortic endothelial dysfunction in diet‐induced obese mice, Kawade 548–559.

PAID

Validity of the short‐form five‐item Problem Area in Diabetes questionnaire as a depression screening tool in type 2 diabetes mellitus patients, Tay 1128–1135.

Pancreas transplant

Diabetes technology including automated insulin delivery systems to manage hyperglycemia in a failing pancreatic graft: Case series of people with type 1 diabetes and a pancreas kidney or pancreas‐only transplant, Stathi 917–920.

Pancreatic cancer

Association of dipeptidyl peptidase‐4 inhibitor use and risk of pancreatic cancer in individuals with diabetes in Japan, Kubota 67–74.

Pancreatic cancer

Association of dipeptidyl peptidase‐4 inhibitor use and risk of pancreatic cancer in individuals with diabetes in Japan, Kubota 67–74.

Pancreatic ductal adenocarcinoma

Diabetes mellitus impacts on expression of DNA mismatch repair protein PMS2 and tumor microenvironment in pancreatic ductal adenocarcinoma, Pan 132–144.

Pathophysiology

Contribution of animal models to diabetes research: Its history, significance, and translation to humans, Yagihashi 1015–1037.

Pemafibrates

Prospective randomized comparative study of the effect of pemafibrate add‐on or double statin dose on small dense low‐density lipoprotein‐cholesterol in patients with type 2 diabetes and hypertriglyceridemia on statin therapy, Hirano 1401–1411.

Peripheral neuropathies

Immediate effect of textured insoles on the balance in patients with diabetic neuropathy, Alaee 435–440.

PHQ9

Validity of the short‐form five‐item Problem Area in Diabetes questionnaire as a depression screening tool in type 2 diabetes mellitus patients, Tay 1128–1135.

PMS2

microenvironment in pancreatic ductal adenocarcinoma, Pan 132–144.

Podocyte injury

LncRNA TCF7 contributes to high glucose‐induced damage in human podocytes by up‐regulating SEMA3A via sponging miR‐16‐5p, Jiang 193–204.

Polar vasculosis

Polar vasculosis is associated with better kidney outcome in type 2 diabetes with biopsy‐proven diabetic kidney disease: A multicenter cohort study, Shimizu 1268–1278.

Polyneuropathies

Associations of polyneuropathy with risk of all‐cause and cardiovascular mortality, cardiovascular disease events stratified by diabetes status, Kim 1279–1288.

Polypharmacy

Effectiveness of countermeasure for polypharmacy by multidisciplinary team review in patients with diabetes mellitus, Nishida 1202–1208.

Posterior reversible encephalopathy syndrome

Pontine lesion in hyperglycemic crises: Relevance to osmotic demyelination syndrome and posterior reversible encephalopathy syndrome, Murao 486–488.

Post‐hoc analysis

Effect of patient characteristics on the efficacy and safety of imeglimin monotherapy in Japanese patients with type 2 diabetes mellitus: A post‐hoc analysis of two randomized, placebo‐controlled trials, Hagi 1101–1109.

Post‐stroke impairment

Association between diabetes mellitus and post‐stroke cognitive impairment, Lee 6–11.

Precision medicine

Progress in genetics of type 2 diabetes and diabetic complications, Shojima 503–515.

Prediabetes

Association of cardiovascular health metrics with annual incidence of prediabetes or diabetes: Analysis of a nationwide real‐world database, Okada 452–462.

Prediabetes and the risk of lung cancer incidence and mortality: A meta‐analysis, Shen 1209–1220.

Elevated serum uric acid is a risk factor for progression to prediabetes in Japanese women: A 5‐year retrospective cohort study, Shimodaira 1237–1245.

Prediabetic state

Comparative efficacy of different eating patterns in the management of type 2 diabetes and prediabetes: An arm‐based Bayesian network meta‐analysis, Zeng 263–288.

Predictive model

Value of machine learning algorithms for predicting diabetes risk: A subset analysis from a real‐world retrospective cohort study, Mao 309–320.

Predictor

Predictive values of metabolic score for insulin resistance on risk of major adverse cardiovascular events and comparison with other insulin resistance indices among Chinese with and without diabetes mellitus: Results from the 4C cohort study, Pan 961–972.

Pregnant women

Advantages of sensor‐augmented insulin pump therapy for pregnant women with type 1 diabetes mellitus, Imafuku 1383–1390.

PROMINENT trial

The influence of triglycerides on small dense low‐density lipoprotein cholesterol levels is attenuated in low low‐density lipoprotein‐cholesterol range: Implications for the negative results of the PROMINENT trial, Hirano 902–906.

Progeria syndrome

A subtype of laminopathies: Generalized lipodystrophy‐associated progeroid syndrome caused by LMNA gene c.29C > T mutation, Huang 1221–1225.

Progesterone

Progesterone levels associated with proteinuria in male diabetes mellitus patients: A cross‐sectional retrospective study, Liu 669–674.

Prognosis

Association of vitamin D status with all‐cause mortality and outcomes among Chinese individuals with diabetic foot ulcers, Weiwei Tang 122–131.

Protein C

A case of fulminant type 1 diabetes and protein C deficiency complicated by deep vein thrombosis, Kohata 1,005–1,008.

Protein load

Robust increase in glucagon secretion after oral protein intake, but not after glucose or lipid intake in Japanese people without diabetes, Ichikawa 1172–1174.

Psoriasis

Weight loss improves inflammation by T helper 17 cells in an obese patient with psoriasis at high risk for cardiovascular events, Maezawa 1136–1139.

Psychometric

Validity of the short‐form five‐item Problem Area in Diabetes questionnaire as a depression screening tool in type 2 diabetes mellitus patients, Tay 1128–1135.

Quantitative sensory testing

Carotid atherosclerosis: An independent risk factor for small fiber nerve dysfunction inpatients with type 2 diabetes mellitus, Guo 289–296.

Renal impairment

Electrocardiogram abnormalities and renal impairment in patients with type 2 diabetes mellitus: A healthcare facilities‐based cross‐sectional study in Dang district of Nepal, Khanal 602–613.

Resistin

Resistin G–A haplotype at SNP‐420/−358 is associated with the latent sarcopenic obesity index in the toon genome study, Ikeda 686–694.

Risk factor

Elevated serum uric acid is a risk factor for progression to prediabetes in Japanese women: A 5‐year retrospective cohort study, Shimodaira 1237–1245.

Safety

Safety of sodium–glucose cotransporter 2 inhibitors in Asian type 2 diabetes populations, Davidson 167–182.

Sandwich ELISA

A newly developed glucagon sandwich ELISA is useful for more accurate glucagon evaluation than the currently used sandwich ELISA in subjects with elevated plasma proglucagon‐derived peptide levels, Kobayashi 648–658.

Sarcopenia

Changes in glycemic control and skeletal muscle mass indices after dapagliflozin treatment in individuals with type 1diabetes mellitus, Yoshimura 1175–1182.

Decreased serum musclin concentration is independently associated with the high prevalence of sarcopenia in Chinese middle‐elderly patients with type 2 diabetes mellitus, Fu 1412–1418.

Sarcopenic obesity

Resistin G–A haplotype at SNP‐420/−358 is associated with the latent sarcopenic obesity index in the toon genome study, Ikeda 686–694.

Second primary cancer

Impact of coexisting diabetes on survival and risk of developing second primary cancer in diabetes patients receiving drug therapy: A multicenter retrospective cohort study of patients with cancer in Japan, Kuwabara 329–338.

Selenoprotein P

Circulating selenoprotein P levels predict glucose‐lowering and insulinotropic effects of metformin, but not alogliptin: A post‐hoc analysis, Takeshita 230–235.

Self‐stigma

Association of self‐stigma with glycated hemoglobin: A single‐center, cross‐sectional study of adults with type 1 diabetes in Japan, Hamano 479–485.

Semaglutide

Effects of switching from liraglutide to semaglutide or dulaglutide in patients with type 2 diabetes: A randomized controlled trial, Iijima 774–781.

Sensor‐augmented pump

Successful introduction of sensor‐augmented pump therapy in a patient with diabetes and needle phobia: A case report, Sugai 1318–1320.

Advantages of sensor‐augmented insulin pump therapy for pregnant women with type 1 diabetes mellitus, Imafuku 1383–1390.

Seroconversion

A surge in serum mucosal cytokines associated with seroconversion in children at risk for type 1 diabetes, Harrison 1092–1100.

Serum cytokine

A surge in serum mucosal cytokines associated with seroconversion in children at risk for type 1 diabetes, Harrison 1092–1100.

Signature metabolites

Ultra‐high performance liquid chromatography coupled to tandem mass spectrometry‐based metabolomics study of diabetic distal symmetric polyneuropathy, Xu 1110–1120.

Simultaneous pancreas–kidney transplantation

Noninvasive evaluation of donor and native pancreases following simultaneous pancreas–kidney transplantation using positron emission tomography/computed tomography, Murakami 1187–1191.

Sinomenine

Sinomenine alleviates diabetic peripheral neuropathic pain through inhibition of the inositol‐requiring enzyme 1 alpha–X‐box binding protein 1 pathway by downregulating prostaglandin‐endoperoxide synthase 2, Chen 364–375.

Skeletal muscle

Blockade of glucagon increases muscle mass and alters fiber type composition in mice deficient in proglucagon‐derived peptides, Ueno 1045–1055.

Skeletal muscle mass

Changes in glycemic control and skeletal muscle mass indices after dapagliflozin treatment in individuals with type 1diabetes mellitus, Yoshimura 1175–1182.

Sleep duration

Sleep duration and food intake in people with type 2 diabetes mellitus and factors affecting confectionery intake, Akiyama 716–724.

Sleep quality

Associations of sleep quality with the skeletal muscle strength in patients with type 2 diabetes with poor glycemic control, Hayashi 801–810.

Slowly progressive insulin‐dependent diabetes mellitus

Comparison of positive rates between glutamic acid decarboxylase antibodies and ElisaRSR™ 3 Screen ICA™ in recently obtained sera from patients who had been previously diagnosed with slowly progressive type 1 diabetes, Takehana 856–863.

Small dense low‐density lipoprotein

The influence of triglycerides on small dense low‐density lipoprotein cholesterol levels is attenuated in low low‐density lipoprotein‐cholesterol range: Implications for the negative results of the PROMINENT trial, Hirano 902–906.

Prospective randomized comparative study of the effect of pemafibrate add‐on or double statin dose on small dense low‐density lipoprotein‐cholesterol in patients with type 2 diabetes and hypertriglyceridemia on statin therapy, Hirano 1401–1411.

Small fiber neuropathy

Carotid atherosclerosis: An independent risk factor for small fiber nerve dysfunction inpatients with type 2 diabetes mellitus, Guo 289–296.

Sodium–glucose cotransporter 2

Luseogliflozin and caloric intake restriction increase superoxide dismutase 2 expression, promote antioxidative effects, and attenuate aortic endothelial dysfunction in diet‐induced obese mice, Kawade 548–559.

Sodium–glucose cotransporter 2 inhibitor

Changes in glycemic control and skeletal muscle mass indices after dapagliflozin treatment in individuals with type 1diabetes mellitus, Yoshimura 1175–1182.

Sodium–glucose cotransporter 2 inhibitors

Real‐world risk of cardiovascular diseases in patients with type 2 diabetes associated with sodium–glucose cotransporter 2 inhibitors in comparison with metformin: A propensity score‐matched model analysis in Japan, Horii 1262–1267.

Safety of sodium–glucose cotransporter 2 inhibitors in Asian type 2 diabetes populations, Davidson 167–182.

Cardiometabolic risk reductions in patients with type 2 diabetes mellitus newly treated with a sodium–glucose cotransporter 2inhibitor versus a dipeptidyl peptidase‐4 inhibitor: A real‐world administrative database study in Japan, Kashiwagi 404–416.

Empagliflozin is associated with lower risk of cardiovascular events and all‐cause mortality in routine care in East Asia: Results from the EMPRISE study, Kim 417–428.

Sox9

Linagliptin ameliorated cardiac fibrosis and restored cardiomyocyte structure in diabetic mice associated with the suppression of necroptosis, Adhikari 844–855.

Special Research Report

A consensus statement from the Japan Diabetes Society: A proposed algorithm for pharmacotherapy in people with type 2 diabetes, Bouchi 151–164.

Spinal cord microglia

Sinomenine alleviates diabetic peripheral neuropathic pain through inhibition of the inositol‐requiring enzyme 1 alpha–X‐box binding protein 1 pathway by downregulating prostaglandin‐endoperoxide synthase 2, Chen 364–375.

Stressful life events

Association of stressful life events with cognitive impairment in patients with type 2 diabetes mellitus, Wang 792–800.

Sulfonylurea

Incretin hormone responses to carbohydrate and protein/fat are preserved in adults with sulfonylurea‐treated KCNJ11 neonatal diabetes, Bowman 1378–1382.

Suramin

Suramin prevents the development of diabetic kidney disease by inhibiting NLRP3inflammasome activation in KK‐Ay mice, Oda 205–220.

Synaptosomal‐associated protein of 29 kDa (SNAP29)

SNARE‐binding protein synaptosomal‐associated protein of 29 kDa (SNAP29) regulates the intracellular sequestration of glucose transporter 4 (GLUT4) vesicles in adipocytes, Matsui 19–27.

Systematic review

Characteristics of subjects with type 2 diabetes enrolled in randomized controlled trials and non‐randomized controlled trials in Japan: A systematic review, Kadowaki 236–246.

T2DM

Validity of the short‐form five‐item Problem Area in Diabetes questionnaire as a depression screening tool in type 2 diabetes mellitus patients, Tay 1128–1135.

TCF7

LncRNA TCF7 contributes to high glucose‐induced damage in human podocytes by up‐regulating SEMA3A via sponging miR‐16‐5p, Jiang 193–204.

Th17

Weight loss improves inflammation by T helper 17 cells in an obese patient with psoriasis at high risk for cardiovascular events, Maezawa 1136–1139.

Time in range

Association between low‐carbohydrate diets and continuous glucose monitoring‐derived time in ranges, Osugi 659–668.

Transcription factor 7‐like 2 gene

Clinical signs of type 1 diabetes are associated with type 2 diabetes marker transcription factor 7‐like 2 polymorphism, Ergur 221–229.

Trend

Trends in incidence rates of childhood type 1diabetes mellitus: A retrospective study in Isfahan province, Iran, Hashemipour 376–386.

Triglyceride‐glucose index

Association of the triglyceride‐glucose index with subclinical left ventricular dysfunction in type 2 diabetes mellitus patients: A retrospective cross‐sectional study, Sun 953–960.

Triglycerides

The influence of triglycerides on small dense low‐density lipoprotein cholesterol levels is attenuated in low low‐density lipoprotein‐cholesterol range: Implications for the negative results of the PROMINENT trial, Hirano 902–906.

Type 1 diabetes

Metabolic signatures of β‐cell destruction in type 1 diabetes, Noso 48–57.

Comparing the clinical significance and antigen specificity of insulinoma‐associated antigen‐2 autoantibodies between radioimmunoassay and enzyme‐linked immunosorbent assay in Japanese patients with type 1 diabetes, Kawasaki 58–66.

Clinical signs of type 1 diabetes are associated with type 2 diabetes marker transcription factor 7‐like 2 polymorphism, Ergur 221–229.

Changes of HbA1c variability after the switch to a longer‐acting insulin analog in people with type 1 diabetes, Watanabe 259–262.

Association of self‐stigma with glycated hemoglobin: A single‐center, cross‐sectional study of adults with type 1 diabetes in Japan, Hamano 479–485.

New‐onset type 1 diabetes and Graves' disease after antiretroviral therapy in a patient with human immunodeficiency virus infection, Taguchi 489–493.

Improving diagnostic accuracy of 3 Screen ICA ELISA kit in the identification of Japanese type 1 diabetes, Kawasaki 1081–1091.

Effect of patient characteristics on the efficacy and safety of imeglimin monotherapy in Japanese patients with type 2 diabetes mellitus: A post‐hoc analysis of two randomized, placebo‐controlled trials, Hagi 1101–1109.

Urinary epidermal growth factor levels correlate with cardiovascular autonomic neuropathy indices in adults with type 1 diabetes, Lin 1183–1186.

Diabetic ketoacidosis due to a sensor defect of FreeStyleLibre: A case report, Kogai 1321–1324.

Type 1 diabetes mellitus

Trends in incidence rates of childhood type 1diabetes mellitus: A retrospective study in Isfahan province, Iran, Hashemipour 376–386.

Graft failure after allogeneic islet transplantation in a patient with type 1 diabetes and a high anti‐glutamic acid decarboxylase antibody titer, Kodani 725–729.

Advantages of sensor‐augmented insulin pump therapy for pregnant women with type 1 diabetes mellitus, Imafuku 1383–1390.

Type 2 diabetes

Sex differences in the association between visceral adipose tissue and atherosclerosis in type 2 diabetes patients with normal bodyweight: A study in a Chinese population, Xu 92–101.

Safety of sodium–glucose cotransporter 2 inhibitors in Asian type 2 diabetes populations, Davidson 167–182.

Characteristics of subjects with type 2 diabetes enrolled in randomized controlled trials and non‐randomized controlled trials in Japan: A systematic review, Kadowaki 236–246.

Progress in genetics of type 2 diabetes and diabetic complications, Shojima 503–515.

Cognitive protection of incretin‐based therapies in patients with type 2 diabetes mellitus: A systematic review and meta‐analysis based on clinical studies, Chai 864–873.

Prospective randomized comparative study of the effect of pemafibrate add‐on or double statin dose on small dense low‐density lipoprotein‐cholesterol in patients with type 2 diabetes and hypertriglyceridemia on statin therapy, Hirano 1401–1411.

Type 2 diabetes mellitus

Carotid atherosclerosis: An independent risk factor for small fiber nerve dysfunction inpatients with type 2 diabetes mellitus, Guo 289–296.

Electrocardiogram abnormalities and renal impairment in patients with type 2 diabetes mellitus: A healthcare facilities‐based cross‐sectional study in Dang district of Nepal, Khanal 602–613.

Sleep duration and food intake in people with type 2 diabetes mellitus and factors affecting confectionery intake, Akiyama 716–724.

Association of stressful life events with cognitive impairment in patients with type 2 diabetes mellitus, Wang 792–800.

Associations of sleep quality with the skeletal muscle strength in patients with type 2 diabetes with poor glycemic control, Hayashi 801–810.

Association of the triglyceride‐glucose index with subclinical left ventricular dysfunction in type 2 diabetes mellitus patients: A retrospective cross‐sectional study, Sun 953–960.

Validity of the short‐form five‐item Problem Area in Diabetes questionnaire as a depression screening tool in type 2 diabetes mellitus patients, Tay 1128–1135.

Efficacy, safety and tolerability of imeglimin in patients with type 2 diabetes mellitus: A meta‐analysis of randomized controlled trials, Hagi 1246–1261.

Machine learning for the prediction of atherosclerotic cardiovascular disease during 3‐year follow up in Chinese type 2 diabetes mellitus patients, Ding 1289–1302.

Association between hemoglobin glycation index and non‐alcoholic fatty liver disease in the patients with type 2 diabetes mellitus, Wang 1303–1311.

Undiagnosed diabetes mellitus

Association of acute‐to‐chronic glycemic ratio and outcomes in patients with COVID‐19 and undiagnosed diabetes mellitus: A retrospective nationwide cohort study, Uchihara 623–629.

Decreased serum musclin concentration is independently associated with the high prevalence of sarcopenia in Chinese middle‐elderly patients with type 2 diabetes mellitus, Fu 1412–1418.

Uric acid

Maternal uric acid levels and risk of gestational diabetes mellitus: A systematic review and dose–response meta‐analysis of cohort studies including 105,380 participants, Nikparast 973–984.

Elevated serum uric acid is a risk factor for progression to prediabetes in Japanese women: A 5‐year retrospective cohort study, Shimodaira 1237–1245.

Validation

Validation and application of the 2019 International Working Group on the Diabetic Foot risk stratification for diabetic foot in Chinese patients, Hu 893–901.

Visceral adipose tissue

Sex differences in the association between visceral adipose tissue and atherosclerosis in type 2 diabetes patients with normal bodyweight: A study in a Chinese population, Xu 92–101.

Vitamin D_3_

Non‐classical monocytes frequency and serum vitamin D_3_ levels are linked to diabetic foot ulcer associated with peripheral artery disease, Hammad 1192–1201.

Weight variability

Effect of intensive lifestyle intervention on the association between weight variability and major adverse cardiovascular events in overweight or obese adults with type 2 diabetes mellitus, Zhong 441–451.

WHO PEN

Baseline prevalence of hyperglycemia and its predictors among community clinic users of a selected rural area of Bangladesh: A cross‐sectional study using the WHO PEN Protocol 1, Faruque 1368–1377.

Wound healing

Local low‐frequency vibration accelerates healing of full‐thickness wounds in a hyperglycemic rat model, Haba 1356–1367.

Yes‐associated protein

Long non‐coding ribonucleic acid zinc finger E‐box binding homeobox 1 antisense RNA 1 regulates myocardial fibrosis in diabetes through the Hippo–Yes‐associated protein signaling pathway, Wu 940–952.

Zinc finger E‐box binding homeobox 1 antisense 1

Long non‐coding ribonucleic acid zinc finger E‐box binding homeobox 1 antisense RNA 1 regulates myocardial fibrosis in diabetes through the Hippo–Yes‐associated protein signaling pathway, Wu 940–952.

